# Intelligence

**Published:** 1827-08

**Authors:** 


### INTELLIGENCE.

### MONTHLY REPORT OF PREVALENT DISEASES.

We have nothing of interest to detail on the present occasion. The weather has been so moderate with regard to heat, as scarcely to give us the usual diseases of the season; and, although Cholera has been met with it, it has been much less frequent than usual.—We have seen more cases of acute inflammation of the respiratory organs than we ever remember to have met with before at the same period of the year.

*Balsam of Copaiba.*—Sir, We live in an age of revivals of old valuable medicines in new forms,—of long-invented instruments restored in varied, but not always improved, shapes: it would be lost time to enumerate every thing of this kind with which the medical public have been entertained for many years. In your last Number (340,) there are two notices on the article Balsam Copaiba, one by Dr. La Roche, the other by Mr. Thorn. I shall confine myself principally to the remarks of the latter gentleman. The preparation of Bals. Copaiba recommended by Mr. T. is an old one presented to the public under a new shape. In the year 1780, on my becoming the pupil of the late ingenious Mr. Wyhe, of Brosley, in Shropshire, among a great variety of preparations he was in the habit of directing to be made in his elaboratory (a most laudable plan), was the evaporation of Balsam Copaiba, on as moderately heated a sand bath as would produce the slowest separation of the essential oil, constantly stirring the mass with a wooden instrument: when it had acquired a soft pilular consistence, it was removed to cool, and potted up like the extracts, and labelled Bals. Copaib. inspissat. I have used it ever since, and always found it a very valuable medicine; nor can I recollect an instance where it proved offensive to the most delicate stomach. Since the recommendation of Cubebs, the union of the two certainly improves both, especially when prescribed for gleets, leucorrhœa, &c. where the case is suitable, as well as some other weakening discharges from the genital organs of both sexes. Joined to Rhubarb and Opium, it is no less efficient in intestinal debilities of long standing. The like may be said of the cases narrated by Dr. La Roche; but, in my own practice, it has afforded me the most steady satisfaction when combined with carefully prepared Ext. Conii in the form of pill, and exhibited with a strong decoction of the Rad. Pareir. Brav. in those cases of foul discharges from the urinary organs connected with irritation of the kidneys, teazing pains, simulation of calculus where none can be found, and all those symptoms which so often perplex the prescriber and harrass the sufferer, without our being able to classify the malady under any ancient or modern nosological designation.

On mentioning the Ext. Conii, I have said "carefully prepared," because I know from long experience that, unless pharmaceutical operators will condescend to inspect the preparation of all delicate articles that come under their notice, they must, when prescribing such medicines, expect to be oftener disappointed than gratified with the result.

I remain, Sir, your obliged friend,

G. N. HILL.

*Chester; June 12, 1827.*

---

*St. George's Hospital.*—Some time ago a pamphlet was published by Mr. Sleigh, (just after his second unsuccessful canvass for the office of surgeon,) containing sundry weighty charges against St. George's Hospital. As Mr. Sleigh's letter was extensively circulated and copied into certain papers, whose zeal induced them to give publicity to the accusations against the hospital, but has not extended so far as to lead them to take any notice of their refutation, we think it right to fill up the blank by laying before our readers the result of a minute enquiry into the grounds on which the charges rested. The investigation was conducted by the following noblemen and gentlemen:—Earl Manvers, Earl Talbot, Earl of Brownlow, Earl of Rosebery, Earl of Clarendon, Earl of Falmouth, Lord Yarborough, Lord Gwydyr, Lord Robert Seymour, the Hon. P. Pusey, the Hon. B. Bouverie, M.P. Wilbraham Egerton, Esq. M.P. George G. Cholmondeley, Esq. John Pepys, Esq. Dr. Seymour, Dr. Gregory, and Dr. Locock; from whose Report we give the following extracts:—

"Your Committee being composed partly of Parliamentary and partly of Professional Members owe it to themselves to state, that they have made a point of devoting four hours in each alternate day, from their first meeting, to the duties of this enquiry; and that, if their Report should appear to present less perfect and conclusive results than might have been contemplated, they confidently ground their apology, not only upon the difficulties incidental to investigations of this peculiar nature, *but upon the course taken by Mr. Sleigh in the prosecution of his charges, which has rendered those difficulties almost insuperable.*

"Your Committee have to remind the Governors, that the printed statement of Mr. Sleigh ascribes to the Weekly Board, and to the attendants of the Hospital, 'a profligate' and 'jobbing' mismanagement for 'interested objects.'

"He infers this from comparisons with other Hospitals, and then details the extravagance under distinct heads, imputing motives, by the words above quoted, for such extravagance.

"Mr. Sleigh did, indeed, early declare to your Committee, that he withdrew all intention of proving these imputations, and confined himself to the simple charge of extravagance.

"And here your Committee cannot but observe, that it will be for the public to judge *how far he may excuse himself for their circulation in print, upon the grounds of mere suspicion, or of the zeal which he alledges for the interests of the poor:* but your Committee have thought it their duty to go beyond the limits thus proposed by Mr. Sleigh for their enquiries, and to investigate all the charges and imputations made by him, as will be seen by the evidence, and by their accompanying resolutions thereon.

"In regard to the single charge of extravagance, Mr. Sleigh's whole proofs have consisted in comparisons with other Hospitals, more particularly with the Middlesex Hospital; and, although invited to assist the Committee in procuring testimony of a more positive nature, both at the outset of their labours, when he altogether declined so doing, and at the close of his remarks upon the evidence adduced, he has still declined adopting the suggestion of your Committee in this respect, unless upon terms which they could not, in the execution of their duty to the governors and public, feel justified in sanctioning.

"Upon this point, the Minutes and Resolutions will best shew both Mr. Sleigh's proposal and the reply which it received from your Committee.

"To found even a tolerably just presumption upon the species of evidence to which Mr. Sleigh has confined himself, it would be necessary not only that the number of patients and amount of expenses should be compared, but that in parity of establishments, mode of keeping accounts, rules of admission and dismissal; in the nature of the cases generally received; in treatment, diet, medical and surgical attendance; in the excellence, and consequent cost, of drugs (which greatly varies according to quality); in the degrees of cure or relief; in the practice, above all, of different and equally eminent physicians; and in a variety of other perplexing yet essential circumstances: the two Hospitals compared, should closely assimilate, if not entirely coincide.

"Your Committee also desire to point out, that material fallacies may exist in any comparison of cost made between two Hospitals. For instance, if the expenses be tried by the number of patients, and not by the beds constantly occupied in both Hospitals, ten beds occupied by four successive sets of patients in a year, averaging three months, would give forty patients; but if by twelve successive sets of patients, averaging a month each, this would give 120.

"The number of beds, therefore, constantly occupied can alone form a tolerable comparison; and here, again, the quantity and cost of medicine used must vary with the nature and severity of disease, as well as the opinions and practice of medical men. The cost of lint at one of the Hospitals, compared by Mr. Sleigh, is six times as much as at St. George's Hospital. The cost of sarsaparilla varies even more the other way: and yet a laborious investigation has accounted for this, without any proof of blame attaching to either Hospital.

"Your Committee have however endeavoured to arrive at a correct conclusion through all these difficulties and details. They have given the closest attention to the object of their appointment, and they entertain the confident hope, that whatever may be the opinion of the Governors as to the manner in which their duty has been discharged, it will not be doubted that, duly conscious of the importance of their investigation to the public, to the governors, and to the poor, they have zealously studied in their examination of witnesses, and in the course of comparison insisted upon by Mr. Sleigh, to elicit, minutely and accurately, whether this long-established Institution does, or does not, deserve the charge of mismanagement made against it in his printed pamphlet.

"They have now to subjoin the following Resolutions, unanimously passed, upon each of Mr. Sleigh's statements :

"Resolved, That in the charge brought against this hospital, in regard to drugs, no evidence whatever has been adduced to prove any fraud, corruption, or negligence, either in the purchase or application of drugs: on the contrary, that they appear to have been bought on the most minute and careful investigation, and on the best principles of competition in the tenders, and of subsequent examination; and administered by the present physicians and surgeons of the hospital with great care, benevolence, and zeal, both for the credit of the hospital and the benefit of their patients. That the criterion of numerical statements of patients and expences in different establishments involves so many difficulties, and requires so much scrutiny, correction, and explanation, that it affords no conclusive evidence of good or bad management, though such statements have been received by the Committee, to assist them in their examination, and to enable them to come to any conclusion which may be beneficial to the hospital.

"That Mr. Sleigh, having more particularly directed his arguments to the existing differences in expence between this and the Middlesex Hospital, your Committee have made as accurate a comparative statement, showing the expences of both, as can be obtained.

| | | | |
|---|---|---|---|
| "The expences in drugs at St. George's Hospital ………… | £1160 | 0 | 0 |
| " Middlesex Hospital ………………………………… | 670 | 0 | 0 |
| "Excess of expenditure of St. George's Hospital over the Middlesex ………………………………………… | 490 | 0 | 0 |

"It is to be observed, that the principal expence of drugs is in administering to in-patients.

"There are twenty in-patients more, daily, in St. George's Hospital, constantly under treatment, than in the Middlesex.

"At the Middlesex Hospital there is a venereal ward, containing fifteen beds, generally full : now these cases may be presumed to consume little medicine. The same may be applied to the cancer ward, which contains ten beds, constantly full. It appears that the out-patients at St. George's Hospital constantly under treatment, exceed those at the Middlesex; but it is impossible to compare the exact number, for the following reasons :

"1st. Out patients do not come regularly for medicine, varying from two-thirds to one-half in number of those actually on the books.

"At the Middlesex Hospital it is the rule, though perhaps not strictly followed, to renew the letters of out-patients every three months, thus multiplying the whole numbers in any given time.

"The physicians and surgeons of St. George's Hospital employ large quantities of sarsaparilla in practice; the medical officers of the Middlesex very little.

"Hence, to meet the surplus of ……………………… £490 0 0

"Twenty in-patients' beds constantly under treatment.

"The excess of sarsaparilla used at St. George's (180*l.*)

"The excess of out-patients.

"The correction of the expences on the venereal and cancer wards.

"The number of accidents, which, in the year under consideration (1825), exceeded those brought into the Middlesex Hospital by 166.

"It having been alleged by Mr. Sleigh, that the vouchers have been wilfully destroyed at this hospital, it appears that the bills of parcels are usually destroyed immediately after having been carefully examined and passed by an annual Committee of Audit, but that the summary of the bills and all the receipts are carefully preserved.

"That the Committee are further of opinion, that Mr. Sleigh's remark with regard to vouchers having been wilfully destroyed, is not warranted by evidence; inasmuch as such vouchers have been produced and passed at the annual Committees of Audit at this hospital.

"Resolved, That no evidence of corruption or negligence in the purchase of surgical instruments, or of abuse in their disposal, has been adduced.

"That the proper expenditure in this article must, in the greatest degree, be confided to the care and judgment of those to whose department this article belongs: remarking, moreover, that the use of any but the very best instruments, kept in the most perfect order, would not only shamefully aggravate the sufferings of the afflicted patients in this hospital, but likewise would induce an indifference in young practitioners to the perfection of their instruments, of the most injurious and cruel nature, and, in consequence, throughout the practice of surgery.

"In regard to any judgment on the expence of surgical instruments in this hospital, as founded on comparison with other hospitals, it appears that the number of accidents in St. George's exceeds those of the Middlesex by upwards of one hundred annually.

[The other Resolutions are equally favourable to the hospital; but, as they relate to matters not strictly professional, we do not conceive it necessary to trouble our readers with them. The extract which we have given is perfectly decisive, and could not be strengthened by any comment of ours.]

---

### NOTICES.

*London University.*—The following professors have been appointed:

Botany and Vegetable Physiology—William Jackson Hooker, LL.D. F.R.S. F.L.S. Professor of Botany in the University of Glasgow.

Zoology—Robert E. Grant, M.D. F.R.S.E. F.L.S.

Anatomy and Physiology, Morbid and Comparative Anatomy, and Surgery—Charles Bell, Esq. F.R.S. F.L.S. Professor to the Royal College of Surgeons; John Frederick Mockel, M.D. Professor of Anatomy and Physiology in the University of Halle in Saxony; Granville Sharp Pattison, Esq. late Professor of Anatomy and Surgery in the University of Maryland, United States.

Nature and Treatment of Diseases.—F. Conolly, M.D.

Midwifery, and the Diseases of Women and Children—David D. Davis, M.D. M.R.S.L.

Materia Medica and Pharmacy—Anthony Todd Thomson, M.D. F.L.S.

*College of Surgeons.*—Sir Astley Cooper has been elected president, and Sir Anthony Carlisle and H. L. Thomas, Esq. vice-presidents for the year ensuing.

*London Hospital.*—Three assistant physicians, and a like number of assistant surgeons, are to be appointed to this hospital.

*St. Bartholomew's Hospital.*—Mr. Abernethy has resigned the office of surgeon to this hospital.

### METEOROLOGICAL JOURNAL,

*From June 20th, to July 20th,* 1827.

By Messrs. HARRIS and Co. Mathematical Instrument Makers, 50, High Holborn.

| June | Rain gauge. | Moon. | Thermom. | | | Barometer. | | De Luc's Hygrom. | | Winds. | | Atmospheric Variations. | | |
|---|---|---|---|---|---|---|---|---|---|---|---|---|---|---|
| | | | 9 A.M. | MAX. | MIN. | 9 A.M. | 10 P.M. | 9 A.M. | 10 P.M. | 9 A.M. | 10 P.M. | 9 *a.m.* | 2 *p.m.* | 10 *p.m.* |
| 20 | | | 61 | 69 | 52 | 29.72 | 29.76 | 80 | 80 | WSW | WSW | Cloudy | Fair | Fine |
| 21 | .5 | | 58 | 68 | 50 | 29.77 | 29.83 | 73 | 77 | WSW | WSW | Fair | Rain | — |
| 22 | | | 58 | 66 | 55 | 29.89 | 29.97 | 77 | 74 | W | W | — | Fair | Fair |
| 23 | | | 60 | 66 | 52 | 29.98 | 30.01 | 76 | 72 | NW | WNW | — | — | Fine |
| 24 | | ● | 60 | 69 | 57 | 29.99 | 29.99 | 73 | 73 | SE | ESE | — | — | — |
| 25 | | | 59 | 67 | 56 | 29.99 | 29.95 | 78 | 80 | ENE | ESE | — | Cloudy | Fair |
| 26 | | | 61 | 72 | 58 | 29.93 | 29.90 | 77 | 74 | WSW | WSW | — | Fair | — |
| 27 | | | 62 | 69 | 58 | 29.83 | 29.66 | 80 | 90 | S | SSW | Cloudy | — | Cloudy |
| 28 | | | 60 | 64 | 58 | 29.53 | 29.57 | 98 | 93 | SW | SW | Rain | Cloudy | Rain |
| 29 | .40 | | 61 | 70 | 58 | 29.52 | 29.62 | 98 | 80 | W | SW | — | — | Cloudy |
| 30 | .6 | | 63 | 72 | 59 | 29.67 | 29.77 | 80 | 80 | WSW | SW | Fair | Rain | — |
| July 1 | | | 62 | 70 | 58 | 29.65 | 29.73 | 88 | 82 | SSE | SW | Cloudy | Cloudy | — |
| 2 | .3 | ☽ | 59 | 71 | 57 | 29.77 | 29.69 | 92 | 88 | S | SW | Rain | Rain | — |
| 3 | | | 62 | 72 | 55 | 29.72 | 29.95 | 82 | 72 | W | WSW | Fair | Fair | Fair |
| 4 | .6 | | 62 | 73 | 62 | 30.06 | 30.06 | 77 | 92 | WSW | WSW | — | Cloudy | Rain |
| 5 | | | 62 | 71 | 55 | 30.10 | 30.31 | 95 | 82 | NE | ENE | Cloudy | Fair | Fine |
| 6 | | | 63 | 71 | 61 | 30.32 | 30.26 | 79 | 82 | S | W | Fair | — | — |
| 7 | | | 66 | 79 | 63 | 30.26 | 30.25 | 78 | 78 | W | N | — | Fine | Fair |
| 8 | | ○ | 66 | 79 | 63 | 30.25 | 30.21 | 74 | 73 | NNW | N | — | — | Fine |
| 9 | | | 71 | 80 | 59 | 30.17 | 30.10 | 71 | 72 | W | WNW | Fine | — | — |
| 10 | | | 75 | 78 | 59 | 30.05 | 29.91 | 76 | 78 | WNW | WNW | — | — | — |
| 11 | | | 60 | 71 | 56 | 29.93 | 30.02 | 82 | 76 | N | NNE | Overcast | — | — |
| 12 | | | 63 | 73 | 57 | 30.05 | 30.07 | 74 | 78 | ENE | E | Fair | — | — |
| 13 | | | 68 | 72 | 54 | 30.07 | 30.09 | 70 | 70 | SE | E | — | — | — |
| 14 | | | 62 | 72 | 53 | 30.07 | 30.01 | 73 | 75 | ENE | ESE | — | — | — |
| 15 | | ☾ | 64 | 74 | 56 | 29.96 | 29.93 | 81 | 74 | NE | ENE | — | — | Cloudy |
| 16 | | | 64 | 72 | 56 | 29.93 | 29.94 | 79 | 78 | E | SSE | — | — | Fair |
| 17 | | | 61 | 76 | 60 | 29.94 | 29.91 | 81 | 75 | S | SW | — | — | — |
| 18 | | | 65 | 73 | 58 | 29.87 | 29.93 | 82 | 69 | SW | W | Cloudy | Fair | — |
| 19 | .35 | | 61 | 69 | 60 | 29.90 | 29.73 | 79 | 95 | WSWv | SW | Sl. Rain | Rain | Rain |

The quantity of Rain fallen in the month of June, was 73.100ths of an inch.

